# Quantifying Transmission of Highly Pathogenic and Low Pathogenicity H7N1 Avian Influenza in Turkeys

**DOI:** 10.1371/journal.pone.0045059

**Published:** 2012-09-18

**Authors:** Roberto A. Saenz, Steve C. Essen, Sharon M. Brookes, Munir Iqbal, James L. N. Wood, Bryan T. Grenfell, John W. McCauley, Ian H. Brown, Julia R. Gog

**Affiliations:** 1 Department of Applied Mathematics and Theoretical Physics, University of Cambridge, Cambridge, United Kingdom; 2 Animal Health and Veterinary Laboratories Agency, United Kingdom; European Union/World Organisation for Animal Health/Food and Agriculture Organization Reference Laboratory for Avian Influenza and Newcastle Disease, Addlestone, Surrey, United Kingdom; 3 Institute for Animal Health, Compton Laboratory, Compton, Newbury, Berkshire, United Kingdom; 4 Department of Veterinary Medicine, University of Cambridge, Cambridge, United Kingdom; 5 Department of Ecology and Evolutionary Biology, Princeton University, Princeton, New Jersey, United States of America; 6 Division of Virology, MRC National Institute for Medical Research, The Ridgeway, Mill Hill, London, United Kindom; University of Oxford, Viet Nam

## Abstract

Outbreaks of avian influenza in poultry can be devastating, yet many of the basic epidemiological parameters have not been accurately characterised. In 1999–2000 in Northern Italy, outbreaks of H7N1 low pathogenicity avian influenza virus (LPAI) were followed by the emergence of H7N1 highly pathogenic avian influenza virus (HPAI). This study investigates the transmission dynamics in turkeys of representative HPAI and LPAI H7N1 virus strains from this outbreak in an experimental setting, allowing direct comparison of the two strains. The fitted transmission rates for the two strains are similar: 2.04 (1.5–2.7) per day for HPAI, 2.01 (1.6–2.5) per day for LPAI. However, the mean infectious period is far shorter for HPAI (1.47 (1.3–1.7) days) than for LPAI (7.65 (7.0–8.3) days), due to the rapid death of infected turkeys. Hence the basic reproductive ratio, 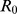 is significantly lower for HPAI (3.01 (2.2–4.0)) than for LPAI (15.3 (11.8–19.7)). The comparison of transmission rates and 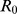 are critically important in relation to understanding how HPAI might emerge from LPAI. Two competing hypotheses for how transmission rates vary with population size are tested by fitting competing models to experiments with differing numbers of turkeys. A model with frequency-dependent transmission gives a significantly better fit to experimental data than density-dependent transmission. This has important implications for extrapolating experimental results from relatively small numbers of birds to the commercial poultry flock size, and for how control, including vaccination, might scale with flock size.

## Introduction

In recent years, outbreaks of avian influenza in poultry have both caused immense economic loss, and continued to pose threats to human health. In 1999–2000 in Northern Italy, an epidemic of a highly pathogenic avian influenza (HPAI) H7N1 virus caused outbreaks in hundreds of farms, predominantly turkey farms, resulting in the death or culling of over 13 million birds [1]. The emergence of HPAI was preceded by outbreaks of low pathogenicity avian influenza (LPAI) virus of the same subtype in more than 199 flocks over the preceding ten months [2]. Though technically defined as low pathogenicity, the LPAI virus was more pathogenic in turkeys than in chickens [3], and in 1999 caused mortality in flocks of meat turkeys ranging from 5% to 97% [2]. A phylogenetic analysis confirmed that the HPAI virus had indeed descended from the LPAI virus over the course of the 1999–2000 outbreaks [4].

An accurate quantitative understanding of the dynamics of spread of avian influenza within the farm level is vital for developing and assessing possible control measures. Arguably, the key epidemiological parameter is 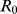, the basic reproduction ratio, the mean number of secondary cases from an infected individual in an otherwise susceptible population [5]. For the H7N1 HPAI virus from the outbreaks in Italy in 2000, attempts have been made to estimate both 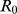 and the transmission rate based on daily mortality data from within farms [6], and similarly for H7N7 HPAI virus in the Netherlands in 2003 [7] and also H5N1 HPAI virus from the 2004 epidemic in Thailand [8]. However, though these studies are hugely valuable, they all face the challenge that the available data are only mortality time series, and thus the whole infection history of birds prior to death must be entirely reconstructed. The estimated parameters are highly sensitive to the assumed duration of infection, leaving a wide range of plausible values for 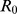. Studies of within farm transmission of LPAI virus face an additional challenge: a lower resolution of data is available than for HPAI, and the precision of final-size analysis based on serosurveillance data will be heavily constrained by sample size [9]. Similarly for analysis of farm to farm transmission, while the high levels of surveillance for the HPAI phase of the H7N1 epidemic means the data are of sufficient quality to enable analysis of between-farm transmission [10], [11], the same approach is not possible for an LPAI phase. Thus a comparison between LPAI and HPAI has not been possible with studies based on empirical data from outbreaks.

Experimental transmission studies offer an alternative approach to characterise the basic infection dynamics, in which a small number of birds are initially infected, and are then co-housed with uninfected contact birds, with swabs taken on a regular basis to assess infection status of each bird. These are challenging experiments to carry out, but they allow independent exploration of virus strain, host species, and the full shedding history of individual birds may be tracked, not just the mortality data. Previous studies have completed transmission studies with H7N7 HPAI in chickens [12] and in turkeys [13], H5N1 [14] and H5N2 [15] HPAI and LPAI in chickens and also H7N1 LPAI in chickens [16]. In many of the results to date, there is great uncertainty in the values of the estimated parameters, for example extremely wide ranges for the confidence intervals for 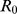 [12], [16]. In addition to this, it is not clear how to extrapolate from parameters derived from experiments (maximum 5 uninfected birds initially per experiment in the studies cited above) to the scale of farm outbreaks.

In this study, we use results from experimental transmission studies of H7N1 in turkeys combined with epidemiological models to estimate transmission rates, the infectious period, and 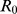. By starting from a single infected bird, and a much larger group of contact birds, the infection dynamics can be determined with greater precision than in previous studies. In addition, by varying the size of the contact group, we can distinguish between competing hypotheses about how infection rates scale with flock size, a key process for understanding transmission dynamics [17]. Experiments were conducted for both LPAI and HPAI viruses of H7N1 subtype, both derived from the outbreaks in Italy in 1999–2000. The experimental host type was turkeys, corresponding to the dominant host species in these outbreaks [1].

## Methods

### Experimental Methods

All animal experiments were carried out under UK Home Office project licence number 70/5015 (‘research into avian viruses and mammalian influenza’) and approved by the local animal ethical committee at AHVLA. All experiments were carried out under SAPO-4 level containment at AHVLA and approved by DEFRA and the Health and Safety Executive.

Individual turkeys (British United Turkeys) at three weeks of age were inoculated intranasally with approximately 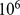 EID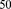 (0.1ml volume) of H7N1 avian influenza virus of either a highly pathogenic (A/ostrich/Italy/984/00, referred to hereafter as HP) or a low pathogenicity (A/chicken/Italy/1279/99, LP). Each inoculated turkey was then immediately housed with a group of uninfected contact birds where the number of contact birds varied by experiment (Table 1), and the enclosure size also was set in proportion to the initial number of birds to maintain contact opportunity. Buccal swabs were taken at intervals (usually daily, details in Table 1) and viral RNA levels were determined by RRT-PCR [18]. A total of five transmission experiments were carried out, three using HP with 10, 20 and 40 contact birds, and two using LP with 40 and 41 contact birds. A summary of the experimental arrangements is given in Table 1, and below experiments are referred to by the strain and number of inoculated and contact birds (e.g. HP 1+20).

**Table 1:** Overview of experiments.

Strain	Inoculated + Contacts Turkeys	Swab Times
HP	1+10	Daily to 5 days
	1+20	Daily to 7 days
	1+40	Daily to 7 days
LP	1+40	Daily to 21 days, except day 4
	1+41	Daily to 18 days, plus 2.5, 3.5 days

Each ropone.0045059.g004.tifw corresponds to one transmission experiment, giving the strain used and the number of inoculated and contact birds. The inoculated birds were given a dose of HP or LP and thereafter housed with the contact birds. The last column lists the times post inoculation of swabs.

### Modelling Methods

The well-established SIR mathematical model of infection dynamics has hosts transitioning from (S)usceptible, to (I)nfected, to (R)ecovered or (R)emoved through death or other means. For the infection process, the standard assumption is that force of infection (the rate that an individual bird becomes infected) is proportional to either the absolute number or the proportion of other hosts currently infected. These are termed density-dependent or frequency-dependent mass action, and represented by 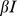 and 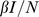 respectively, where 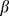 is the transmission rate, 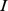 is the number of currently infected birds, and 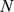 is the total number of birds currently housed [5]. These two variants are indistinguishable when 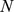 is constant, but behave differently when the total number of birds can vary, for example through removal by death or when comparing parameters across experiments with different numbers of birds. Both models were considered for all experiments, and also for HP combined and LP combined where we fitted a shared parameter across the LP or HP experiments.

A continuous time stochastic infection model was used for the transmission process between each consecutive pair of observation time points. Generally these time intervals were one day, but for the 1+41 LP experiment some observations were 12 hours apart, and in the 1+40 LP experiment a missing day was handled as a single two-day transition. Recoveries and deaths were not included in the individual transition probability calculations, so the resulting model within each transition is a pure SI model, and we compute the likelihoods of the observed number of new infections given the initial number of susceptibles and infecteds (see for example [19]). The product of these over all the transitions gives the overall likelihood for the transmission rate 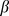 for each experiment (or combined experiments).

In the simplest theoretical models, the recovery or removal rate is a constant parameter throughout infection, which leads to exponentially distributed infectious periods. In practice however, this is often a very poor fit to observed distributions of infectious periods [5] as is the case in the present study. Here, a general gamma distribution was used to compute likelihoods based on the observed periods of shedding. It was assumed that the infection start time was uniformly distributed in the interval between the first positive swab and the previous (negative) swab, and that the infection ended between the last positive swab, and the next observation point (death or a negative swab).

The basic reproductive ratio 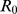 here is simply the population mean infectious period multiplied by the transmission rate. A profile likelihood function for the population mean infectious period is calculated, i.e. maximising the likelihood function over gamma distribution parameters that yield a given mean. In turn, a profile likelihood function for 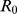 is calculated by the similar method, based on the likelihood functions for the transmission rate and mean infectious period. For all parameters, the estimates given below are maximum likelihood point estimates (MLE) [20]. The 95% confidence intervals (CIs), were computed by determining the range where the profile log likelihood for the parameter of interest was within 1.92 logs from the maximum, corresponding to a chi-square distribution with one degree of freedom.

## Results

### Summary of Transmission Experiments

The status of each turkey at each time point in each of the five experiments is given in Tables 2, 3, 4, 5, 6. A bird was considered infectious if viral RNA was detected on swabs by PCR, and considered recovered if at least two consecutive samples were negative for viral RNA. Accordingly, some samples were inferred to be false positives or false negatives in order to make a logical infection history (with a single continuous period of shedding).

**Table 2:** Transmission of HP in 1 inoculated and 10 contact turkeys (HP 1+10).

Turkey	Day post infection					
	0	1	2	3	4	5
0	–	+	+	x		
1	–	–	+	+	x	
2	–	–	–	+	x	
3	–	–	–	+	x	
4	–	–	–	+	x	
5	–	–	–	+	x	
6	–	–	–	+	x	
7	–	–	–	+	+	x
8	–	–	–	+	+	x
9	–	–	–	+	+	x
10	–	–	–	+	+	x

Turkey 0 was inoculated with HP on day 0 as described in methods, and then co-housed with 10 naïve in-contact turkeys. Table symbols are as follows: +, positive buccal swab; –, negative buccal swab; x, bird died or killed. In this and subsequent tables, for ease of reading the rows for the contact turkeys are arranged in order of first positive swab and then subsequent negative swab.

**Table 3:** Transmission of HP in 1 inoculated and 20 contact turkeys (HP 1+20).

Turkey	Day post infection							
	0	1	2	3	4	5	6	7
0	–	+	x					
1	–	–	+	x				
2	–	–	+	+	x			
3	–	–	+	+	x			
4	–	–	–	+	x			
5	–	–	–	+	x			
6	–	–	–	+	x			
7	–	–	–	+	+	x		
8	–	–	–	+	+	x		
9	–	–	–	+	+	x		
10	–	–	–	–	x			
11	–	–	–	–	+	x		
12	–	–	–	–	+	x		
13	–	–	–	–	+	x		
14	–	–	–	–	+	(+)	x	
15	–	–	–	–	+	(+)	x	
16	–	–	–	–	+	(+)	x	
17	–	–	–	–	+	(+)	+	x
18	–	–	–	–	–	–	x	
19	–	–	–	–	–	–	x	
20	–	–	–	–	–	–	+	x

Turkey 0 was inoculated with HP on day 0 as described in methods, and then co-housed with 20 naïve in-contact turkeys. Table symbols are as follows: +, positive buccal swab; –, negative buccal swab; x, bird died or killed; (+) buccal swab negative, but bird inferred to be shedding to make a logical infection history.

**Table 4:** Transmission of HP in 1 inoculated and 40 contact turkeys (HP 1+40).

Turkey	Day post infection							
	0	1	2	3	4	5	6	7
0	–	–	x					
1	–	–	x					
2	–	–	+	x				
3	–	–	+	+	x			
4	–	–	+	+	x			
5	–	–	+	+	x			
6	–	–	+	+	x			
7	–	–	+	+	x			
8	–	–	+	+	x			
9	–	–	+	+	+	x		
10	–	–	+	+	+	x		
11	–	–	+	+	+	x		
12	–	–	–	+	x			
13	–	–	–	+	x			
14	–	–	–	+	x			
15	–	–	–	+	x			
16	–	–	–	+	x			
17	–	–	–	+	+	x		
18	–	–	–	+	+	x		
19	–	–	–	+	+	x		
20	–	–	–	+	+	x		
21	–	–	–	+	+	x		
22	–	–	–	+	+	x		
23	–	–	–	+	+	x		
24	–	–	–	+	+	x		
25	–	–	–	+	(+)	+	x	
26	–	–	–	+	+	+	x	
27	–	–	–	–	x			
28	–	–	–	–	x			
29	–	–	–	–	x			
30	–	–	–	–	+	x		
31	–	–	–	–	+	x		
32	–	–	–	–	+	x		
33	–	–	–	–	+	x		
34	–	–	–	–	+	x		
35	–	–	–	–	+	x		
36	–	–	–	–	+	+	x	
37	–	–	–	–	+	+	x	
38	–	–	–	–	+	+	+	x
39	–	–	–	–	–	x		
40	–	–	–	–	–	x		

Turkey 0 was inoculated with HP on day 0 as described in methods, and then co-housed with 20 naïve in-contact turkeys. Table symbols are as follows: +, positive buccal swab; –, negative buccal swab; x, bird died or killed; (+) buccal swab negative, but bird inferred to be shedding to make a logical infection history.

**Table 5:** Transmission of LP in 1 inoculated and 40 contact turkeys (LP 1+40).

Turkey		Day post infection																				
	0	1	2	3	4	5	6	7	8	9	10	11	12	13	14	15	16	17	18	19	20	21
0	–	+	+	+	nt	+	x															
1	–	+	+	+	nt	+	x															
2	–	+	+	+	nt	+	+	+	(+)	+	–	–	–	(−)	–	–	–	–	–	–	–	–
3	–	–	+	+	nt	+	+	+	+	+	+	(+)	x									
4	–	–	+	+	nt	+	+	+	+	+	+	+	+	+	–	–	–	–	–	(−)	–	–
5	–	–	–	+	nt	+	+	+	+	+	–	–	–	–	nt	nt	nt	nt	nt	nt	nt	nt
6	–	–	–	+	nt	+	(+)	+	+	+	+	–	–	–	–	–	(−)	–	–	(−)	–	–
7	–	–	–	+	nt	+	+	+	+	nt	+	–	–	–	–	–	–	–	–	(−)	–	–
8	–	–	–	+	nt	+	+	+	+	+	+	–	–	(−)	(−)	–	(−)	–	–	–	–	–
9	–	–	–	+	nt	+	+	+	+	+	+	–	–	(−)	–	–	(−)	(−)	–	–	–	–
10	–	–	–	+	nt	+	+	+	+	+	+	–	–	(−)	–	–	(−)	–	–	–	–	–
11	–	–	–	+	nt	+	+	+	+	+	+	–	–	(−)	–	–	(−)	–	–	–	–	–
12	–	–	–	+	nt	+	+	+	+	+	+	–	–	(−)	–	–	(−)	–	–	–	–	–
13	–	–	–	+	nt	+	+	+	+	+	+	–	–	–	(−)	–	(−)	(−)	–	–	–	–
14	–	–	–	+	nt	+	+	+	+	+	+	–	–	–	–	–	(−)	–	–	–	–	–
15	–	–	–	+	nt	+	+	+	+	+	+	–	–	–	–	–	–	–	–	–	–	–
16	–	–	–	+	nt	+	+	+	+	+	+	–	–	–	–	–	–	–	–	–	–	–
17	–	–	–	+	nt	+	+	+	+	+	+	–	–	–	–	–	–	–	–	–	–	–
18	–	–	–	+	nt	+	+	+	+	+	+	+	–	–	–	–	–	–	–	–	–	–
19	–	–	–	+	nt	+	+	+	+	+	+	+	(+)	+	–	–	(−)	–	–	–	–	–
20	–	–	–	+	nt	+	+	+	+	+	+	nt	(+)	+	nt	(+)	+	–	–	–	–	–
21	–	–	–	–	nt	+	+	+	x													
22	–	–	–	–	nt	+	+	+	+	+	x											
23	–	–	–	–	nt	+	+	+	+	+	x											
24	–	–	–	–	nt	+	+	+	+	+	+	x										
25	–	(−)	–	–	nt	+	+	+	+	+	+	–	–	–	–	(−)	(−)	–	–	–	–	–
26	–	–	–	–	nt	+	(+)	+	+	+	+	–	–	(−)	–	–	–	–	–	–	–	–
27	–	–	–	–	nt	+	(+)	+	+	+	+	–	–	–	–	–	–	–	–	–	–	–
28	–	–	–	–	nt	+	+	+	+	+	+	–	–	(−)	(−)	(−)	(−)	–	–	(−)	(−)	–
29	–	–	–	–	nt	+	+	+	+	+	+	–	–	(−)	–	–	–	–	–	–	–	–
30	–	–	–	–	nt	+	+	+	+	+	+	–	–	(−)	(−)	–	(−)	–	–	–	–	–
31	–	–	–	–	nt	+	+	+	+	+	+	–	–	–	(-)	–	–	–	–	–	–	–
32	–	–	–	–	nt	+	+	+	+	+	+	–	–	–	–	–	(−)	–	–	–	–	–
33	–	–	–	–	nt	+	+	+	+	+	+	–	–	–	–	(−)	(−)	–	(−)	(−)	–	–
34	–	–	–	–	nt	+	+	+	+	+	+	–	–	–	–	–	–	–	–	–	–	–
35	–	–	–	–	nt	+	+	+	+	+	+	–	–	–	–	–	–	–	–	–	–	–
36	–	–	–	–	nt	+	+	+	+	+	+	–	–	–	–	–	–	–	–	–	–	–
37	–	–	–	–	nt	+	+	+	+	+	+	+	(+)	+	–	–	–	–	–	–	–	–
38	–	–	–	–	nt	+	+	+	+	+	+	(+)	+	+	–	–	(−)	–	–	–	–	–
39	–	–	–	–	nt	–	+	+	+	+	+	–	–	–	–	–	(−)	–	–	–	–	–
40	–	–	–	–	nt	–	+	+	+	+	+	–	–	–	x							

Turkey 0 was inoculated with LP on day 0 as described in methods, and then co-housed with 40 naïve in-contact turkeys. Table symbols are as follows: +, positive buccal swab; –, negative buccal swab; x, bird died or killed; (+) buccal swab negative, but bird inferred to be shedding to make a logical infection history; (−) buccal swab positive but bird inferred to have recovered to make a logical infection history; nt, not tested.

**Table 6:** Transmission of LP in 1 inoculated and 41 contact turkeys (LP 1+41).

Turkey		Day post infection																			
	0	1	2	2.5	3	3.5	4	5	6	7	8	9	10	11	12	13	14	15	16	17	18
0	−	+	+	+	+	+	x														
1	−	−	+	+	+	+	+	+	+	+	x										
2	−	−	+	+	+	+	+	+	+	+	+	+	x								
3	−	−	+	+	+	+	+	+	+	+	+	+	+	−	−	−	−	−	−	−	−
4	−	−	+	+	+	+	+	+	+	+	+	+	+	−	−	−	−	−	−	−	−
5	−	−	+	+	+	+	+	+	+	+	+	+	+	(+)	x						
6	−	−	+	+	+	+	(+)	+	+	+	+	+	+	(+)	+	−	−	−	−	−	−
7	−	−	+	+	+	+	+	+	+	+	+	+	+	(+)	+	(+)	+	+	−	−	−
8	−	−	−	+	+	+	+	+	x												
9	−	−	−	+	+	+	+	+	+	+	+	+	+	+	+	(+)	+	−	−	−	(−)
10	−	−	−	+	+	+	+	+	+	+	+	+	+	(+)	+	(+)	+	+	−	−	−
11	−	−	−	−	+	+	+	x													
12	−	−	−	−	+	+	+	+	x												
13	−	−	−	−	+	+	+	+	+	x											
14	−	−	−	−	+	+	+	+	+	x											
15	−	−	−	−	+	+	+	+	+	x											
16	−	−	−	−	+	+	+	+	+	+	+	x									
17	−	−	−	−	+	+	+	+	+	+	+	x									
18	−	−	−	−	+	+	+	+	+	+	+	+	x								
19	−	−	−	−	+	+	+	+	+	+	+	+	+	x							
20	−	−	−	−	+	+	+	+	+	+	+	nt	+	−	−	−	(−)	(−)	−	−	−
21	−	−	−	−	+	+	+	+	+	+	+	+	+	−	−	−	−	−	−	−	−
22	−	−	−	−	+	+	+	+	+	+	+	+	+	−	−	−	−	−	−	−	−
23	−	−	−	−	+	(+)	+	+	+	+	+	+	+	+	x						
24	−	−	−	−	+	+	+	+	+	+	+	+	+	+	−	−	−	−	(−)	−	−
25	−	−	−	−	+	+	+	+	+	+	+	+	+	(+)	+	−	−	−	−	−	−
26	−	−	−	−	+	+	+	+	+	+	+	+	+	+	+	−	−	−	−	−	−
27	−	−	−	−	+	+	+	+	+	+	+	+	+	+	+	−	−	x			
28	−	−	−	−	+	+	+	+	+	+	+	+	+	+	+	−	−	−	x		
29	−	−	−	−	+	+	+	+	+	+	+	+	+	+	+	+	−	−	−	−	−
30	−	−	−	−	+	(+)	+	+	(+)	+	+	+	+	+	+	(+)	+	−	−	−	−
31	−	−	−	−	+	+	+	+	+	+	+	+	+	(+)	+	(+)	+	−	−	−	−
32	−	−	−	−	−	+	+	+	x												
33	−	−	−	−	−	+	+	+	+	x											
34	−	−	−	−	−	+	+	+	+	+	x										
35	−	−	−	−	−	+	(+)	+	+	+	+	+	+	+	+	−	−	−	−	−	(−)
36	−	−	−	−	−	+	+	+	+	+	+	+	+	(+)	+	+	+	+	−	−	−
37	−	−	−	−	−	+	+	+	+	+	+	+	+	+	+	+	(+)	+	+	−	−
38	−	−	−	−	−	−	+	+	+	+	x										
39	−	−	−	−	−	−	+	+	+	+	+	+	+	+	+	−	−	−	(−)	−	(−)
40	−	−	−	−	−	−	−	+	+	+	+	+	+	−	−	−	−	−	−	−	−
41	−	−	−	−	−	−	−	+	+	+	+	+	+	+	+	+	+	+	x		

Turkey 0 was inoculated with LP on day 0 as described in methods, and then co-housed with 41 naïve in-contact turkeys. Table symbols are as follows: +, positive buccal swab; −, negative buccal swab; x, bird died or killed; (+) buccal swab negative, but bird inferred to be shedding to make a logical infection history; (−) buccal swab positive but bird inferred to have recovered to make a logical infection history; nt, not tested.

In all five experiments, all inoculated and contact birds became infected (Tables 2, 3, 4, 5, 6). In the experiments with HP, all birds died (73 out of 73), which included two euthanised on welfare grounds (birds 8 and 38 in Table 4). In the LP experiments 30 out of 83 birds died: the remaining birds had at least two consecutive negative swabs before the end of the experiment, indicating recovery from infection.

### Frequency-dependent Versus Density-dependent Transmission

**Figure 1:**
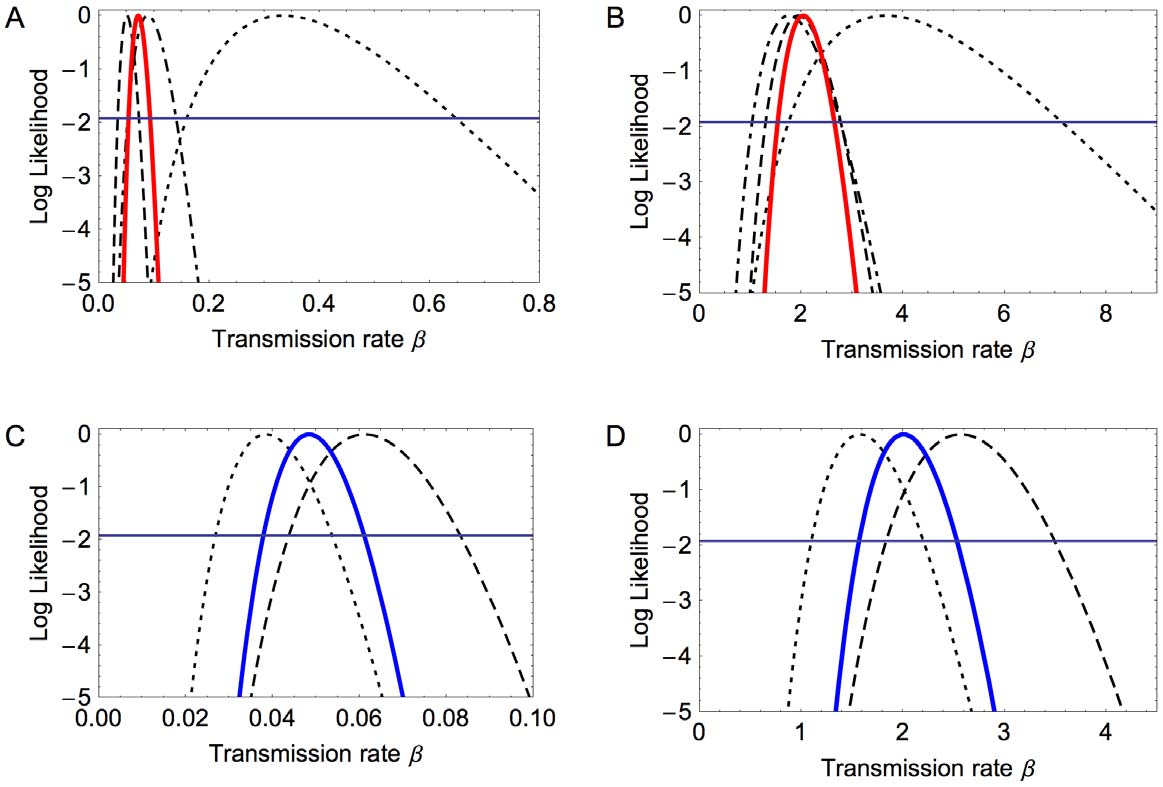
Model fits for transmission rates. A–D show the log likelihoods for the transmission rate. In A and B the curves correspond to the HP experiments: 1+10 (dotted), 1+20 (dash-dotted), 1+40 (dashed), combined (solid red), and C and D are similarly for the LP experiments: 1+40 (dotted), 1+41 (dashed), combined (solid blue). A and C are for the density-dependent transmission model and B and D are for frequency-dependent.

Likelihoods were computed for the transmission rate for both density- and frequency-dependent mass-action, for each experiment and for the HP and LP experiments in combination, see Table 7 and Figure 1. For the LP, the vast bulk of transmission events happened before any deaths, so the models are virtually indistinguishable for individual experiments. Similarly, combining the two experiments using a single parameter does not distinguish the two models (log-likelihood difference of 0.18).

**Table 7:** Transmission Parameters and Comparison of Models.

Strain	Experiment	Density-dependent transmission		Frequency-dependent transmission	
		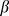 (95% CI)	log-likelihood	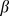 (95% CI)	log-likelihood
HP	1+10	3.34 (1.6–6.5)×10^−1^	–4.44	3.67 (1.8–7.1)	−4.44
	1+20	8.88 (5.2–14.0)×10^−1^	−6.01	1.75 (1.0–2.8)	−5.93
	1+40	5.09 (3.4–7.3)×10^−1^	−4.81	1.94 (1.3–2.8)	−4.33
	Combined	7.15 (5.4–9.3)×10^−1^	−24.34	2.04 (1.5–2.7)	−16.17
LP	1+40	3.85 (2.7–5.4)×10^−1^	−7.97	1.58 (1.1–2.2)	−7.97
	1+41	6.12 (4.4–8.3)×10^−1^	−15.33	2.57 (1.8–3.5)	−15.32
	Combined	4.84 (3.8–6.1)×10^−1^	−25.16	2.01 (1.6–2.5)	−25.34

The maximum likelihood estimates for the transmission rate for both density- and frequency-dependent transmission (units for 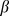 are per bird per day for density-dependent, and per day for frequency-dependent). The 95% CI and the value of the log-likelihood at the maximum are given for each of the experiments separately, and also when fitted across all HP or LP experiments in combination.

For the HP, all the transmission events had occurred before the first death in the smallest experiment (1+10), and the estimates for the transmission rate were rather broad under either model. For the two larger HP experiments, the number of birds was decreasing through death while new transmission events were still happening, but still the difference between the performance of the models (as measured by the maximum likelihood value) was small for each experiment on its own. However, the fitted transmission rates for the density-dependent model varied considerably over the three different-sized experiments, and hence forcing a common transmission parameter across all experiments produces a poor fit. In contrast, the frequency-dependent fitted transmission rates were relatively stable across the three experiments, and the confidence intervals are securely overlapping (Figure 1 panel B). Comparing the likelihoods for the two models for the combined HP experiments: the likelihood is 8.2 logs greater for the frequency-dependent than for the density-dependent model (Table 7). In summary, the data are more consistent with a frequency-dependent mass-action transmission. This form of mass-action corresponds with the number of contacts of each bird remaining constant when the total population size varies.

### Parameter Estimates and Comparison between LP and HP

**Figure 2:**
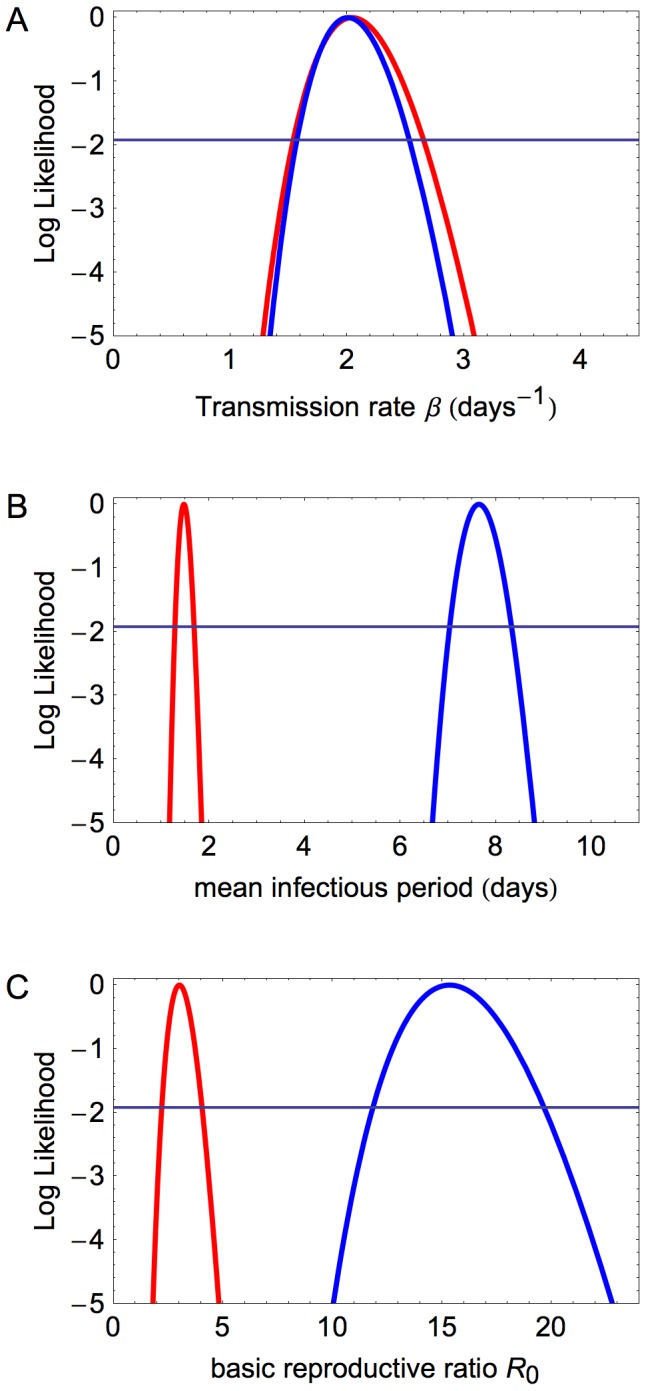
Comparison of parameters for LP and HP. Log likelihoods are shown for (A) the transmission rate, (B) the mean infectious period and (C) the basic reproduction ratio, for LP (blue) and HP (red). The log likelihoods are shifted so that their maxima are at zero, and the horizontal line is at −1.92.

Focussing now only on the preferred frequency-dependent infection model, the fitted transmission rates for HP and LP are shown together in Figure 2 (panel A). The transmissibility of the two strains appear to be indistinguishable: the estimated transmission rate for LP is 2.01 days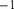 (CI 1.6–2.5) and for HP is 2.04 days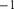 (CI 1.5–2.7).

**Figure 3:**
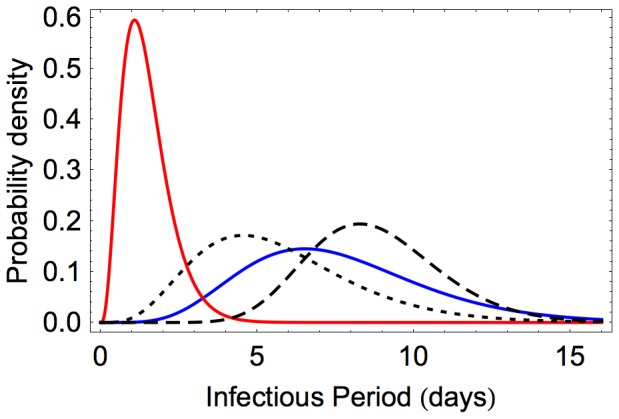
Infectious period distributions. The maximum likelihood gamma distributions for the infectious period are shown for LP (blue) and HP (red). These represent the variability between different birds in duration of shedding. The LP infections were also split into two groups and fitted separately according to whether infection concluded with recovery (dashed) or death (dotted).

The observed infectious periods were not exponentially distributed, so as described above the continuous gamma distribution was fitted (Figure 3). These distributions reflect the variability between birds in their duration of infection, and the profile likelihood functions for the mean infectious period are shown in Figure 2 panel B. The estimate for the mean infectious period for LP was 7.65 days (CI 7.0–8.3), which is longer than that for HP which was 1.47 days (CI 1.3–1.7). For HP, this short infectious period is the result of rapid death of all infected birds. For LP, the fitted mean infectious period for the 30 birds that died was 5.69 days (CI 4.8–6.8). This was shorter than for the 53 birds that recovered, which was 8.78 days (CI 8.2–9.4).

Combining the distributions for the transmission rates and the mean infectious periods, we arrive at a distribution for the basic reproductive ratio, 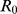 (Figure 2 panel C). These are very clearly different: for HP the maximum likelihood estimate is 3.01 (CI 2.2–4.0), for LP the MLE is 15.3 (CI 11.8–19.7). As the transmission rates for the two strains are very similar, the difference between the strains is almost entirely determined by the infectious period, which in turn is shaped by the probability and timing of disease-induced death. A summary of the fitted parameters and CI ranges are given in Table 8.

**Table 8:** Estimates of key parameters for combined HPAI and LPAI experiments.

Strain	Transmission Rate *β*	Mean Infectious Period	Reproductive Ratio *R_0_*
HP	2.04 (1.5–2.7) day^−1^	1.47 (1.3–1.7) days	3.01 (2.2–4.0)
LP	2.01 (1.6–2.5) day^−1^	7.65 (7.0–8.3) days	15.3 (11.8–19.7)

The maximum likelihood estimates for key parameters for HP and LP experiments combined, with 95% CI in parentheses.

## Discussion

In this study we have provided estimates of the transmission rate, mean infectious period and the basic reproductive ratio for both highly pathogenic and low pathogenicity strains of infection with H7N1 in turkeys. In contrast to previous transmission studies, we have been able to identify narrower confidence intervals for the key parameters. This resolution has been possible through having experiments with only a single initial infected bird and a larger number of contact birds than used before, thus providing a broad window to analyse transmission events.

While daily swabs give a detailed picture of infection status for each bird, model fits are still constrained to the resolution of the experiments: the dynamics are determined only down to the timescale of sampling. The exact time of infection or recovery is unknown, only the time window in which it could have occurred, and so the precise timing must be fitted or assumed. In particular, constraining recovery/death to the observed timepoints means that we may have overestimated the number of infected birds at any given time and thus correspondingly fitted low values of the transmission rates. However, we expect this to only have a small effect on our parameter estimates.

The mean latent period in these experiments is shorter than our observational timescale of a day. For four of the five transmission experiments, the birds initially inoculated were shedding virus by 24 hours, and in the fifth, the swab was negative at 24 hours but by 48 hours the inoculated bird was dead. In a separate experiment with ten turkeys initially inoculated with the same HP strain under the same conditions as this study and swabbed every 8 hours, three birds were shedding by 8 hours and all birds were shedding by 16 hours (data not shown). A previous study had assumed a fixed latent period of 48 hours, though in those data all ten of the unvaccinated inoculated birds were shedding virus by 24 hours, which was the first time point after inoculation [12]. Other studies have attempted to fit the latent period from transmission experiments, though this has not been achieved yet with much accuracy. For H7N1 LPAI in chickens it was identified as less than a day [16] and for H5N1 in chickens the latent period was fitted as less than six hours though with a wide confidence interval [14]. Here we have assumed that the latent period is negligible over the timescales considered. This simplifying assumption is likely only to have very slightly reduced the estimated transmission rates. These estimates could be refined in future by identifying the latent period distribution directly from experimental observations, which would require considerably more frequent testing in the hours following initial inoculation.

Though the number of birds in our experiments appears to be the largest in the literature for experimental analysis of transmission of HPAI, the experimental scale is still much smaller than the scale of interest: a commercial turkey farm. Here, we have explicitly tested the two main competing forms of model for population size dependence on transmission [21] and found frequency-dependent mass-action to be the better-fitting model. In this form, the number of contacts per bird remains constant as flock size changes, meaning that the 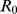 should not depend on flock size. While our results indicate that frequency-dependent mass-action may be operating at least on the scale of the experiments presented here, it is possible that a different process could be operating on a much larger scale. However, analysis of observed outbreaks suggests that frequency-dependence may also hold at the farm scale: studies of within-farm mortality data in both the Italian H7N1 and the Dutch H7N7 epidemics found that estimated transmission rates were not detectably affected by flock size [6], [7]. Even if population size has no effect, it might be predicted that bird density and husbandry type may affect transmission [21]. In the experiments reported in this study, pen sizes were adjusted accord to group size to ensure a consistency in the rate of contact. However, observational data confirmed that the turkeys tended to huddle in a single group, consistent with the level of contact seen in commercial production. Encouragingly, our HP parameters are consistent with the parameter ranges in the study based on within-farm mortality data from the HPAI phase of the H7N1 epidemic [6].

We found transmission rates for LP and HP to be indistinguishable, but the infectious period was far shorter for HP, indicating a lower corresponding 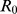 (Table 8). This raises the challenging question of how H7N1 LPAI virus gave way to the HPAI virus in the 1999–2000 epidemic. While the molecular mechanism is known [4], the evolutionary driving force behind selection remains unclear. What our study shows is that the HPAI virus was unlikely to have had a simple within-flock advantage. It is possible that having a shorter latent period would lend an advantage to HPAI over LPAI, as would the additional contribution to infection if dead birds were not removed as assiduously as in an experimental arrangement, but it is hard to see how in practice these effects could come close to outweighing the difference in infectious period in determining within-flock invasion.

Alternative mechanisms for selection of the HPAI virus, beyond a simple within-flock advantage, include of course the possibility that the between-farm transmission risk was higher for HPAI. In addition, our study considers only turkeys. Multiple species were involved in the 1999–2000 epidemic, though turkeys accounted for the majority of infected flocks. While HPAI virus appears to be devastating across a range of species, the LPAI caused more severe pathology in turkeys than chickens [3]. Indeed, a recent study of a H7N1 LPAI strain in chickens found a similar infectious period to that we found in turkeys, but a considerably lower transmission rate [16]. This could be through some combination of chickens being less infectious or less susceptible to the virus. Indeed susceptibility to H5N1 HPAI virus differs between chickens and turkeys [22]. In conclusion, transmission rates and 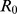 for H7N1 LPAI and HPAI may not follow the same pattern for different species.

Speculatively, prior exposure by the LPAI virus could have conferred some partial protection to HPAI virus, so that birds could be infected but not so quickly killed by the virus. Thus, the H7N1 LPAI outbreaks in 1999 could have laid the groundwork for the successful invasion of the HPAI virus when it emerged. During the incident of H7N7 in Oxfordshire, UK in 2008, in which HPAI virus emerged from LPAI virus on a single farm, HPAI virus spread to poultry sheds housing birds with prior immunity as a result of exposure to the LPAI infection. These birds remained clinically healthy but did shed virus. It is therefore possible that the dynamic in partially immune or immune birds to a LPAI virus to a corresponding HPAI phenotype is such that there will still be infection and shedding, so cryptic infection is possible (Brown, I.H. pers. obs. and [23]).

Our study was able to specify fitted parameters to a greater accuracy than previously possible for either studies based on observed mortality data from outbreaks, or for experimental transmission studies. We made full use of the time series of infection status to fit transmission rates. This approach will be more accurate than using only the information of final size, particularly when the final size in small experimental groups may be all birds infected for even moderate 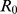 values. In addition, each of the five transmission experiments used in this study was initiated with a single inoculated bird. For a fixed number of birds, this may be expected to give the maximum information about transmission rates, assuming that the first generation of infection does not fail. Finally, the use of varying numbers of birds meant that hypotheses on how infection rates scale with population size could be investigated. We strongly recommend that future studies consider this approach to ensure that results from relatively small experiments may be more securely extrapolated up to commercial poultry farm scale.
